# Transactions of the American Medical Association, Vol. XIV

**Published:** 1864-04

**Authors:** 


### Book Notices.

TRANSACTIONS OF THE AMERICAN MEDICAL ASSOCIATION, VOL. XIV. 1864.

This is a handsomely printed and bound volume of 416 pages; containing the Record of Proceedings; the Address of the President; the Report on Medical Education, by C. C. Cox, M.D., of Maryland; the Report on Medical Literature, by Charles A. Lee, M.D., of New York; a paper on Diatheses: their Surgical Relations and Effects, by E. Andrews, M.D., of Chicago; a paper on the American Method of Treating Joint Diseases and Deformities, by Henry G. Davis, M.D., of New York; Cases of Diarrhœa Adiposa, by John H. Griscom, M.D., of New York; a Report on American Necrology, by C. C. Cox, M.D., of Maryland; a Prize Essay on the Physiological and Medicinal Properties of the Veratrim Veride, &c., by Samuel R. Percy, M.D., of New York; a paper on Laryngoscopal Therapy, &c., &c., by Louis Elsberg, A.M., M.D., of New York; the Constitution and Code of Ethics of the Association, with the List of Officers and Permanent Members. The volume, though not a large as some of its predecessors, contains much matter of very great interest to the Profession. The Prize Essay on Veratrum Viride is, alone, worth twice the cost of the whole book. It is a model paper, and might be read with profit by every practitioner. Our present purpose, however, is simply to announce the publication and enumerate the contents of the work. It is supplied to all members of the Association who have paid the Annual Assessment for the year 1863; and other members of the profession may procure it by forwarding *three dollars* to the Treasurer, Dr. Caspar Wister, of Philadelphia.

